# Management of Advanced and Metastatic Prostate Cancer: A Need for a Sub-Saharan Guideline

**DOI:** 10.1155/2019/1785428

**Published:** 2019-12-05

**Authors:** Ayun Cassell, Bashir Yunusa, Mohamed Jalloh, Medina Ndoye, Mouhamadou M. Mbodji, Abdourahmane Diallo, Saint Charles Kouka, Issa Labou, Lamine Niang, Serigne M. Gueye

**Affiliations:** ^1^Department of Urology and Andrology, Hopital General de Grand Yoff, Dakar, Senegal; ^2^Department of Surgery, Liberia College of Physicians and Surgeons, Monrovia, Liberia; ^3^UFR Sante, Université de Thiès, Thiès, Senegal

## Abstract

The estimated incidence rate of prostate cancer in Africa was 22.0/100,000 in 2016. The International Agency for Research on Cancer (IARC) has cited prostate cancer as a growing health threat in Africa with approximated 28,006 deaths in 2010 and estimated 57,048 deaths in 2030. The exact incidence of advanced and metastatic prostate cancer is not known in sub-Saharan Africa. Hospital-based reports from the region have shown a rising trend with most patients presenting with advanced or metastatic disease. The management of advanced and metastatic prostate cancer is challenging. The available international guidelines may not be cost-effective for an African population. The most efficient approach in the region has been surgical castration by bilateral orchidectomy or pulpectomy. Medical androgen deprivation therapy is expensive and may not be available. Patients with metastatic castrate-resistant prostate cancer tend to be palliated due to the absence or cost of chemotherapy or second-line androgen deprivation therapy in most of Africa. A cost-effective guideline for developing nations to address the rising burden of advanced prostate cancer is warranted at this moment.

## 1. Introduction

Approximately 174,650 new prostate cancer cases were estimated to be diagnosed in 2019 which is about 20% of new cancer cases in men [1]. Evidence has shown that prostate cancer accounted for 9.8% of all cancer-related death in males [2]. In the past few years, in the United States, there has been a slight increase in metastatic prostate cancer and prostate cancer-related mortality, 26,730 deaths in 2017 to 31,620 death in 2019 [1, 2]. According to the 2019 National Comprehensive Cancer Network Guidelines (NCCN), the increase in the number of metastatic prostate cancer may have been influenced by guideline recommendations which could have resulted in reduced detection of localized prostate cancer and lesser radical prostatectomies being performed [1].

The International Agency for Research on Cancer (IARC) highlighted prostate cancer as a growing health threat in Africa with approximated 28,006 deaths from prostate cancer in 2010 and estimated 57,048 deaths in 2030. In sub-Saharan Africa, the Institute for Health Metrics and Evaluation (IHME) study estimated the prostate cancer death to have increase from 5,600 in 1990 to 12,300 in 2010 [3]. A systemic review and meta-analysis by Adeloye et al. in 2016 showed a rise in prostate cancer incidence and mortality in Africa [4]. The estimated pooled prostate cancer incidence rate in the study was 22.0/100,000 with a 95% confidence interval of 19.93–23.97 [4].

The management of prostate cancer is challenged by the late presentation in most part of Africa even though some men will die of other causes than prostate cancer. Contemporary reports from Nigeria, Senegal, and Uganda have shown that most men have limited knowledge of prostate cancer screening [5]. There are no established means of preventing prostate cancer or curing advance disease; therefore, early detection and appropriate management are the best options. Studies have shown that African Americans present with more advanced prostate cancer, have shorter progression-free survival, and were more likely to develop prostate cancer at a younger age and more likely to progress to metastasis before clinical diagnosis [6]. These results can be simply extrapolated for men in the sub-Sahara because of race and genetic similarities.

The exact incidence of advanced and metastatic prostate cancer is not known in sub-Saharan Africa. Hospital-based reports from the region have shown a rising trend with most patients presenting with advanced or metastatic disease. Androgen deprivation therapies are the frequently used treatment modality for patients with advanced disease in the region. Luteinizing hormone-releasing hormone (LHRH) analogues and antiandrogen are expensive for most patients, and surgical castration has been widely adopted in sub-Saharan Countries. Radiotherapy may have a role in the management of locally advanced and advanced disease in Africa, but the use has been limited by the lack of specialist facilities, oncologists, and radiotherapists in the region [4]. Nevertheless, there has been ongoing training and research in areas of radio-oncology and prostate cancer management in Ghana and Senegal [5]. In 2012, the African Organization for Research and Training in Cancer launched the African Cancer Network Project in effort to improve collaboration amongst various institutions working for cancer control. There were approximately 102 cancer centers listed, but most of them were in South Africa [7].

The problem remains with patients with advanced disease who have disease progression following medical or surgical castration as docetaxel-based chemotherapy and second-line antiandrogen medications are either expensive or not available. Some palliative care initiatives have instituted in some parts of Africa by the African Palliative Care Association (APCA) to address pain management in advanced disease and improve the well-being of terminal patients [7].

There is currently no cost-effective prostate cancer guideline for sub-Saharan nations with advanced or metastatic disease. Most of the practices have been extrapolated from international guidelines which are not fully applied due to the associated cost of treatment or availability of recommended regimens. This review projects the challenges of advanced/metastatic prostate cancer and the importance of prostate cancer guidelines for sub-Saharan Africa.

## 2. Methodology

The literature was reviewed from 2000 to 2018 using search engines: PubMed, Google Scholar, African Journal Online, and Google. Both English and French Literature were searched using the term Advanced or Metastatic Prostate Cancer and appended with the following indices: Sub-Saharan Africa, Senegal, Togo, Ivory Coast, Ghana, Togo, Benin, Liberia, Nigeria, Kenya, Burkina Faso, and Mali. There were a total of more than 400 articles. After sorting the publications, only 50 articles were included in the study. These papers included systemic reviews and meta-analysis, randomized controlled trials, clinical guidelines, review articles, and prospective and retrospective studies. The abstracts or full texts from the sub-Saharan literature were reviewed for age, staging presentation, prostate-specific antigen (PSA), and Gleason scores (Table 1), presenting symptoms and site of metastasis (Table 2), and treatment modality and outcome (Table 3).

The American Joint Committee on Cancer (AJCC) TNM staging was used to classify advanced prostate cancer as T3-T4 + N0 or N1 and metastatic as any T, any N, and M1. Only percentages of advanced and metastatic tumors were extracted and reflected in the study (Table 1). A quantitative pooled analysis of the data from 15 sub-Saharan African studies considering parameters as mean age, age range, mean PSA, and PSA range was performed and elaborated in the results. The Gleason scores were restratified into Gleason grade group (1–5) according to the American Urological Association Prostate Cancer guidelines. Qualitative analysis of the presenting symptoms, site of metastasis, treatment modalities, and outcome was done and discussed in the Results section.

## 3. Results

A total of 15 studies from the sub-Saharan region with published literature on advanced prostate cancer were reviewed. A pooled analysis of 2744 patients showed a mean age of 68.7 years (age range of 30 to 100 years) [8–22]. Reports from Burkina Faso, Togo, Nigeria, Kenya, Senegal, and Ivory Coast showed that 67% to 99% of patients managed for prostate cancer presented with advanced or metastatic disease [8–16, 19, 20, 22]. However, Gueye et al. [17] and Yeboah et al. [21] reported a much lower population of advanced and metastatic prostate cancer (41.3%/5.8% and 25%/13%), respectively. The mean PSA was 532.7 ng/ml with a PSA range of 0–21660 ng/ml. Study population that included exclusively advanced and metastatic prostate cancer as published by Diallo et al. [8], Botcho et al. [9], and Ndoye et al. [11] displayed a higher mean PSA (1991.5 ng/ml, 1373.3 ng/ml, and 1447.6, respectively) and PSA range (5–216600 ng/ml). The highest Gleason grade group from the 15 studies reviewed was approximately 44% of Gleason grade groups 2–5, stating that there was a significant amount of moderately to poorly differentiated advanced prostate cancer (Table 1). Subsequently, only 2 published studies from Burkina Faso, Kirakoya et al. [10] and Kaboré et al. [14], reported the highest Gleason grade group as being grade group 1 prostate cancer accounting for 59.9% and 60.1%, respectively.

The commonest presenting symptoms were obstructive lower urinary tract symptoms followed by pain with an average presentation of 60% and 50.6%, respectively [8, 12, 13, 19, 22]. The commonest bony metastatic site in the review was the spine [8, 11–13, 18–20, 22], but Yeboah et al. [21] reported metastasis of 8.6% to the pelvic bone. The lungs were the commonest site of visceral metastasis [8, 10, 11, 21, 22] followed by the liver [10, 12, 20]. On the contrary, data from Badmus and colleagues [19] showed a 54% metastasis to the rectum (Table 2).

In patients with advanced and metastatic prostate cancer, surgical and medical androgen deprivations were the commonest modalities offered [8–10, 12–15, 19, 21–23]. Yeboah et al. [21] clearly emphasized the use of neoadjuvant hormonotherapy, androgen deprivation therapy, external beam radiation, brachytherapy, channeling TURP, and transurethral incision of the prostate as modalities in the management of patients reported for advanced and metastatic prostate cancer. Apparently, the francophone sub-Saharan nations (Senegal, Togo, Burkina Faso, and Ivory Coast) reported cyproterone acetate as the commonly used antiandrogen while Ghana, Nigeria, and Kenya used stilboestrol as an antiandrogen in the management of advanced and metastatic prostate cancer. The use of LHRH analogue, bicalutamide, and flutamide was also reported in most studies; nevertheless, docetaxel-based chemotherapy was shown in only one report from Ghana [21]. The average hospital mortality shown from four studies was about 13.2%. Diallo et al. recorded 8% mCRPC in patients on androgen deprivation therapy. The 5-year overall survival by Ekwere and Egbe [13] and Badmus et al. [19] was 0% and 7.4%, respectively (Table 3).

## 4. Discussion of the Standard Guidelines for Advanced/Metastatic Prostate Cancer

Patients with (cT3-T4 or cN+) any (PSA or Gleason score) are classified as locally advanced prostate cancer [24]. The management of locally advanced prostate cancer remains controversial. Different treatment modalities exist for locally advanced prostate cancer including surgery, radiation therapy, androgen-deprivation therapy, or combination [25–29]. However, the benefit of these modalities against each other has not been validated in prospective randomized trials.

Treatment of prostate cancer recurrence after radical prostatectomy remains controversial. A study by Panebianco and colleagues has demonstrated the efficacy of positron emission tomography and multiparametric magnetic resonance imaging in the detection of prostate cancer recurrence after radical prostatectomy [30]. In the absence of systemic metastasis and rising serum PSA, the disease is considered locoregional recurrence or disease persistence, and salvage radiotherapy could be a first option [30]. When there is evidence of metastasis, radiation to the postprostatectomy bed should be avoided due to morbidity and androgen deprivation therapy should be preferred.

There is currently no consensus on the management of prostate cancer in sub-Saharan Africa as most of the treatment options are directly adopted from international guidelines. Yet, to remain in conformity with these guidelines has been challenging. In 2010, reports from the sub-Saharan region estimated that there were approximately less than two surgeons per 100,000 residents [7]. To a greater extent, most of the oncological surgeries are performed by general surgeons. The Directory of Radiotherapy Centers coordinated by the International Atomic Energy Agency (IAEA) estimated a total of 277 external beam radiotherapy machines present on the African continent, but nearly 60% of these are found in Egypt and South Africa alone [7]. It has been reported that there may be about 22 chemotherapeutic drugs on the World Health Organization Drug List that are brought to Africa at some point in time, but these drugs are rather too expensive and lack the experts for proper administrations [7]. These are barriers to a comprehensive prostate cancer care in the region, and more robust action is required to achieve international standards. However, knowledge of the current guidelines and recommendation is the first step to this journey.

### 4.1. Locally Advanced cT3a Tumor

Clinical T3a tumor could benefit from radical prostatectomy and extended lymph node dissection based on the results from randomized trials [25–29]. This treatment option is proven beneficial because some T2 tumor (13%–27%) could be overstaged to T3 [25–28]. It is difficult to counsel patients for these treatment options as cT3b and cT4 tumors on the contrary have been understaged to cT3a as studies have shown that T2-T3a have a better outcome with radical prostatectomy than T3b-T4 tumors. Nevertheless, cT3a patients selected for radical prostatectomy should be offered adjuvant hormonotherapy or salvage radiotherapy [25–29]. The recent 5- and 10-year overall survival following radical prostatectomy for cT3a tumors ranges from 75% to 97.6% and 60% to 94.8%, respectively [25–28].

### 4.2. Locally Advanced cT3b-T4 Tumors

Evidence has shown survival benefits with radiation therapy and androgen ablation therapy for these groups of patients with prostate cancer [24]. The role of radical prostatectomy in cT3b-T4 tumors remains unclear.

In a prospective study of 669 patients with prostate cancer in Ghana by Yeboah et al. [18], patients with locally advanced prostate cancer (T3/T4) tumors were treated with neoadjuvant hormonotherapy ± total androgen blockade followed by brachytherapy or external beam radiotherapy. Other hormonal treatment included stilboestrol ± aspirin. Patients with locally advanced disease presenting with bladder outlet obstruction were managed with alpha-blockers or finasteride, with some patients requiring a transurethral incision of the prostate (TUIP) or transurethral resection of the prostate (TURP) for symptomatic control. There was a modest response to radiotherapy with some side effects as hematuria, radiation cystitis, proctocolitis, rectal bleeding, and 30.4% disease progression on external beam radiotherapy.

### 4.3. Management of Metastatic Castration-Naïve Prostate Cancer (mCNPC)

The standard of care for mCNPC has been continuous androgen-ablation therapy. Androgen deprivation can be achieved using LHRH analogues, bilateral orchidectomy, or LHRH analogues + first-generation antiandrogen [2]. However, the STAMPEDE and CHAARTED trials have shown an overall survival benefit in high volume (mCNPC) receiving continuous androgen-ablation therapy and docetaxel (chemo-hormonotherapy) [23, 29, 31, 32]. Results from the STAMPEDE and LATITUDE studies also projected a greater median overall survival with abiraterone and prednisone in addition to androgen deprivation therapy for locally advanced and metastatic prostate cancer as compared to ADT alone [33, 34]. Nevertheless, the overall survival has been comparable for chemo-hormonotherapy or abiraterone/prednisolone + ADT according to the results from the STAMPEDE trial [34, 35].

Most African literature studies [8–10, 12–15, 19, 21–23] have documented surgical castration by bilateral pulpectomy or orchidectomy as the preferred treatment option in patients with mCNPC. This approach is cost-effective as most patients in the region cannot afford medical ADT, but the approach remains irreversible. The real challenge lies with patients who have disease progression following surgical castration as chemotherapy (like docetaxel) and second-line ADT (abiraterone or enzalutamide) are expensive or not available. In most clinical scenarios in sub-Saharan Africa, the only available option will be to palliate.

Sow and colleagues [23] in Senegal evaluated 102 patients with metastatic hormone-sensitive prostate cancer and the patients were divided into two groups: one group received ADT alone by either medical or surgical castration while the other group received ADT + cytoreductive therapy in the form of either open prostatectomy or TURP. The results showed that patients receiving ADT + cytoreductive therapy had a better overall survival (median 24 months vs. 14 months, *p*=0.03) and progression-free survival (median 43 months (group 2) vs. 20 months (group 1)) as compared to those on ADT alone. This result could suggest a role of cytoreductive therapy in patients with metastatic prostate cancer, but more data are needed to validate this approach.

### 4.4. Management of Metastatic Castrate-Resistant Prostate Cancer (mCRPC)

Patients presenting with prostate castrate testosterone level <50 ng/dl with either 3 consecutive PSA rise 1 week apart + 50% above the nadir with (PSA >2 ng/ml) [36] or radiological evidence of two or more new bony lesions or enlargement of a soft tissue lesion are considered castrate-resistant [37].

Data from a large phase III trial COU-AA-302 in chemo-naïve patients have shown an acceptable overall survival (OS) and radiological progression-free survival (PFS) with abiraterone + prednisolone as compared to placebo [38]. There was modest hepatotoxicity from mineralocorticoids and abiraterone. Enzalutamide has shown a statistical improvement in overall survival in chemo-naïve mCRPC in a phase III trial (PREVAIL) [39]. The noticeable side effects were hypertension and fatigue. A phase III randomized placebo-controlled trial (AFFIRM) showed some benefit in men with metastatic CRPC who had received prior docetaxel chemotherapy with significant PSA decline, radiographic response, and prolonged time to the first skeletal-related event [2]. Henceforth, enzalutamide is a reasonable treatment option for both predocetaxel and postdocetaxel metastatic CRPC setting and should be considered for men who are not fit for chemotherapy. However, men with nonmetastatic CRPC show some progress on apalutamide based on a phase 3 SPARTAN with improvement of metastasis-free survival over placebo with ADT [2].

A significant improvement in overall survival has been displayed with docetaxel-based chemoregimen as compared to other chemotherapy [36, 40]. Docetaxel is now the first-line chemotherapy for metastatic prostate cancer and administered as 75 mg/m^2^ every 3 weeks combined with prednisone 5 mg BID, up to 10 cycles [36]. Due to the heterogeneous population of mCRPC, prognostic factors to consider include PSA level, PSA density, anemia, visceral metastasis, and bone scan progression [36]. Patients with mCRPC who progress on docetaxel-based regimen can benefit from cabazitaxel, a taxane derivative [36]. Side effects of neutropenia and sepsis are high with this regimen [36, 41]. Evidence has demonstrated that patients with mCRPC who progress on docetaxel chemoregimen, enzalutamide, or abiraterone + prednisone have provided favorable overall median survival as compared to placebo [36].

Sipuleucel-T, an active cellular immunotherapy, has provided some overall survival benefit in mCRPC with no visceral metastasis as compared to placebo in a phase III randomized trial [42]. Its use has been limited by access and cost [36]. The only bone-specific drug radium 223, an alpha emitter, was found to increase median overall survival and prolonged time to the first skeletal event following a large phase III trial (ALSYMPCA) of 921 symptomatic mCRPC patients who were not fit or failed chemotherapy [36].

In a phase III trial conducted by the Cancer and Leukemia Group B (CALGB 9583), a subgroup of patients who had progressed on ADT with rising PSA were treated with ketoconazole + hydrocortisone. About 33% had a PSA decline and 7% had objective response [43]. Though ketoconazole is not a first-line option for mCRPC, it should be considered in the developing settings where other first-line antiandrogens are not available or too expensive. Nevertheless, larger prospective controlled studies are required in Africa to establish an evidence-based efficacy to the current standard of care.

Data from a hospital-based study in Ghana revealed that 4.6% of patients with locally advanced prostate cancer treated with hormonotherapy subsequently developed castrate-resistant prostate cancer, and these patients were managed with stilboestrol, ketoconazole/prednisolone, docetaxel or cyclophosphamide, and prednisolone [21]. However, a report from Senegal by Diallo et al. [8] evaluating patients with metastatic prostate cancer reported 8% of castrate-resistant prostate cancer in patients following hormonotherapy.

### 4.5. Management of Complications of Bony Metastasis

Metastasis of prostate cancer to the bone or spine is associated with bone pain, spinal cord compression, pathological fractures, and vertebral deformities. Patients with spinal cord compression should be treated as an emergency with a high dose of corticosteroid and a neurosurgical consultation for decompression [36, 44]. However, external beam radiation can be used as an alternative for decompression and pain relief [36, 44, 45]. Symptomatic mCRPC patients who are not fit or failed docetaxel therapy can benefit from radium 223 as it is associated with improved overall survival, reduced skeletal complications, and better quality of life [36]. Bisphosphonates have been useful in reducing bone resorption in mCRPC and reducing bone pain [46, 47]. Zoledronic acid 4 mg in mCRPC patients was helpful in reducing pathological fractures and other bone complications as shown in results from controlled studies [46]. Patients on zoledronic acid should have routine dental consultation as the risk of osteonecrosis of the jaw is high [46, 47]. Data have shown that denosumab, a monoclonal antibody, is superior to zoledronic acid in preventing skeletal-related events but showed no difference in the survival benefits between both [36].

The rate of bony metastasis in patients with prostate cancer is relatively high according to most series in the sub-Saharan region [8, 11–13, 18–20, 22]. However, most of these literature studies did not document the available options to treat or palliate bony metastasis. According to the African Palliative Care Association (APCA), a few countries including Uganda have initiated pain control with morphine for patients with advanced cancer at a modest price (Stephan). A study from Ghana [21] documented that 0.9% of patients in the study with bony metastasis received doses of external beam radiotherapy. Data by Kirakoya et al. [10] reported some of the patients with prostate cancer metastasis to spine with associated neurological deficits presented to the neurosurgical department before further urological assessment.

## 5. Follow-Up

The objectives for follow-up in patients with mCRPC are to assess response to treatment or palliation. Regular visits at urology clinics are advisable to assess symptom progression. These visits can reduce the risk of spinal cord compression especially in patients presenting with back pain. Rising serum creatinine can stimulate the need to detect silent ureteral obstruction or chronic urinary retention. PSA should be monitored following initiation of treatment. Evidence has shown that patients in whom PSA nadir remains <2 ng/ml after 5–7 months of follow-up have a better median survival [36]. Reducing hemoglobin with subsequent anemia explains the fatigue in these patients. Elevated liver enzymes may signify disease progression or toxicity that are common with nonsteroidal antiandrogen drugs. The progression of symptoms could stimulate the need for further imaging, but the goal should be to improve the patient's quality of life.

Patients on LHRH analogues should achieve a serum testosterone level at castrate level (<1 nmol/L) [36]. However, 13–38% could have treatment failure; therefore, it is practical to monitor serum testosterone every 3–6 months. Surgical castration should be considered an option in patients who fail medical androgen ablation therapy. Patients on androgen deprivation therapy should be screened for metabolic syndrome and cardiovascular disease. Fasting blood sugar, HbA1c, lipid profile, and consultation with a cardiologist should remain part of the follow-up [36].

The review from the sub-Saharan region showed that the overall 5-year survival ranged from 0% to 5% with an in-hospital mortality of 13.2%. This dismal result could have been as a result of poor follow-up, patients' noncompliance with the treatment protocol, the lack of adequate finance to continue treatment, the lack of access to healthcare, the lack of the proper regimen, or simply overtreatment. However, most studies did not document their outcome and so it is difficult to reach a conclusion.

## 6. Road Map to a Sub-Saharan African Guideline

The success of any guideline in the region will require a multidisciplinary team including urologists, surgical/medical oncologists, radio-oncologists, radiologists, oncology nurses, and researchers. All stakeholders of urological cancer on the continent of Africa should be involved in strategic planning and implementation including the World Health Organization (WHO), International Atomic Energy Agency (IAEA), Pan African Urological Association (PAUSA), international cancer societies, local cancer societies, national urological associations, and pharmaceutical companies.

From contemporary practice, surgical castration has been a major armamentarium for androgen ablation in advanced prostate cancer in most of Africa. However, the use of medical ablation therapy (nonsteroidal: flutamide, bicalutamide, nilutamide, enzalutamide, and apalutamide/steroidal: cyproterone acetate, megestrol acetate, and chlormadinone acetate) and LHRH agonist (leuprolide, goserelin, triptorelin, and histrelin) has been reported in different studies from Africa. Most of these drugs are costly and will require appropriate financing. The continental average total health expenditure per capita is approximately US$82 which is far below what is available in the United Kingdom (US$3,512) [7]. Most African nations have promised to increase health expenditure but yet to be achieved [7].

It is practical that pharmaceutical companies could play an essential role in meeting these objectives. Evidence has shown that only 3% of global drugs are produced in Africa and most being imported from abroad [48]. This has contributed to the high cost of cancer drugs considering the underfunding in the health system already. Only South Africa and Morocco have managed to produce 70% to 80% of their drug supply [48]. Currently, policies should be geared towards local production of essential drugs especially chemotherapeutic agents which are very costly. This could drastically improve the outcome of cancer care in enacted.

To any given point, the use of any therapeutic agent should be driven by either evidence from a clinical research or guideline consensus from a professional group. For example, excerpts from a Prostate Cancer Guideline Panel in Europe proposed a three-step methodological concept to constitute a consensus: (1) a systematic review to describe heterogeneity across studies (2) a two-round Delphi survey involving a large, international panel of stakeholders, including healthcare practitioners (HCPs) and patients; and (3) a consensus group meeting attended by stakeholder group representatives [49]. Their goals were to create consensus statements that would guide clinical practice until further prospective or randomized trials could be conducted.

It will be practical that urologists and oncologists from Africa adopt these models and endorse these into plans during annual conferences and convention. The need for coordinated research and collaboration on guidelines is overdue on the continent. Nevertheless, we remain optimistic for continuous national governmental commitment and support for cancer in the region.

## 7. Conclusion

The management of advanced and metastatic prostate cancer is challenging. The available guidelines may not be cost-effective for an African population. The most efficient approach in the region has been surgical castration by bilateral orchidectomy or pulpectomy. Medical androgen deprivation therapy is expensive and may not be available. Patients with metastatic castrate-resistant prostate cancer tend to be palliated due to the absence or cost of chemotherapy or second-line androgen deprivation therapy in most of Africa. A cost-effective guideline for developing nations to address the rising burden of advanced prostate cancer is warranted at this moment.

## Figures and Tables

**Table 1:** The demographics, biodata, staging presentation, PSA, and Gleason grade group of patients with prostate cancer in sub-Saharan nations.

Study	No. of patients	Mean age in years	Age range in years	Staging presentation	PSA range	Mean PSA	Highest Gleason grade group
Diallo et al. (Senegal) [8]	156	75.3	52–100	Advanced/metastatic	1,300–2,104	1991.5	Group 4 (36%)
Botcho et al. (Togo) [9]	132	71 ± 12.6	45–99	Advanced/metastatic	5–9,998	1373.3 ± 1078	Groups 3–5 (46%)
Kirakoya et al. (Burkina) [10]	82	68.9 ± 9.5	49–95	98.6% (T3/T4) 40.2% metastasis	13–9,224	746	Group 1 (59.7%)
Ndoye et al. (Senegal) [11]	102	71 ± 9	51–96	Advanced/metastatic	5.88–21,660	1447.6 ± 812	Groups 2–5
Tengue et al. (Togo) [12]	232	68.5 ± 9.6		82.9% (T3/T4) 75.9% metastasis		123.5	Groups 2-3 (34.5%)
Ekwere and Egbe (Nigeria) [13]	145	66.6 ± 9.8	35–88	81.4% (T3/T4)			
Kaboré et al. (Burkina) [14]	168	68.59 ± 9.41	30 to 95	86% (T3/T4)	1–7,421	483.3 + 145.4	Group 1 (60.1%)
Wasike and Magoha (Kenya) [15]	65	67	50–100	87.5% (T3/T4)			Group 4 (39.3%)
Kaboré et al. (Burkina) [16]	166	71.5	52–86	73.6% (T3/T4)	8.4–17,850	537	Groups 2-3 (54.7%)
Gueye et al. (Senegal) [17]	121	69	52–88	41.33% (T3/T4) 5.8% metastasis	6–578.9	72.2	
Ikuerowo et al. (Nigeria) [18]	43	60.8		40% (T3/T4) 35% metastasis	0–438.3	2.5	Groups 2–5 (74.4%)
Badmus et al. (Nigeria) [19]	189	68	40–100	94.2% (T3/T4) 91% metastasis		106 ± 187	
Folasire et al. (Nigeria) [20]	82	67 ± 1.8	47–87	Advanced/metastatic			Groups 4-5 (38%)
Yeboah et al. (Ghana) [21]	699	70 ± 0.04	41–94	25% (T3/T4) 13% metastasis	2.5–9,900	52.5	Groups 2–5 (67.7%)
Konan et al. (Ivory coast) [22]	362	67.4	44–97	68.2% metastasis	0–6000	315.0	Groups 2-3 (48.6%)
Sow et al. (Senegal) [23]	102	71.1 ± 8.6	54–88	Metastatic	PSA nadir	1,167.7	Group 3

**Table 2:** The presenting symptoms and site of metastasis being either bony or visceral.

Study	Commonest presenting symptoms	Commonest metastatic sites	
		Bony	Visceral
Diallo et al. [8]	Obstructive LUTS (59.4%)	Spine (551%)	Lungs (14%)
Folasire et al. [20]	Pain (70%)	Spine (94%)	Liver (18%)
Badmus et al. [19]	Obstructive LUTS (82.5%)	Spine (46.1%)	Rectum (54.0%)
Kirakoya et al. [10]	Obstructive LUTS (69.5%)	Spine (47.6%)	Lungs/liver (10.5%)
Yeboah et al. [21]	Obstructive LUTS (16%)	Pelvic bone (8.2%)	Lungs (0.6%)
Ndoye et al. [11]	Pain (31.3%)	Spine (22.5%)	Lungs (13.7%)
Tengue et al. [12]	Obstructive LUTS (89.2%)	Spine/pelvic bone (79%)	Liver (6.8%)
Ekwere and Egbe [13]	Obstructive LUTS (56%)	Spine/ribs	
Ikuerowo et al. [18]		Spine/pelvic bone	
Konan et al. [22]	Obstructive LUTS (47.8%)	Spine (44.5%)	Lungs (13%)

LUTS: lower urinary tract symptoms.

**Table 3:** The available treatment modalities, the medications used, and the outcomes of treatment in some studies.

Study	Available treatment modality	Available hormonotherapy drugs	Outcome
Diallo et al. [8]	Surgical ADT (76.3%), medical ADT (22.4%)	Cyproterone acetate	8% castrate resistant
Botcho et al. [9]	Surgical ADT (38.6%), medical ADT, both (18.9%)	Cyproterone acetate, flutamide, LHRH analogue, chemotherapy	
Tengue et al. [12]	Surgical ADT (34.5%), medical ADT (12.5%), both (46.1%)	Cyproterone acetate, flutamide	15.1% mortality
Kirakoya et al. [10]	Surgical ADT (24.3%), medical ADT (62.2%), both (13.4%)	Cyproterone acetate, LHRH analogue	10.9% mortality
Kaboré et al. [14]	Surgical ADT (43.3%), medical ADT (19%), both (9.5%)	Cyproterone acetate, LHRH analogue	
Wasike and Magoha [15]	Mostly surgical ADT, few medical ADT	Low-dose stilboestrol	
Badmus et al. [19]	Surgical ADT (71.9%), medical ADT (24.3%), both (22.2%)	Stilboestrol, bicalutamide, goserelin, flutamide	5-year overall survival (7.4%)
Yeboah et al. [21]	Neoadjuvant hormonotherapy ± medical ADT, EBRT, brachytherapy, TUIP, TURP	Docetaxel, LHRH analogue, stilboestrol, bicalutamide /flutamide	11% hospital mortality
Ekwere and Egbe [13]	Surgical ADT and medical ADT	Stilboestrol	0% 5-year overall survival
Konan et al. [22]	Surgical ADT (86.2%), medical ADT (13.7%)	Cyproterone acetate, LHRH analogue	16.2% mortality
Sow et al. [23]	Group 1: surgical ADT and medical ADT only Group 2: cytoreductive therapy + castration		Group 1: 6-month median (OS) Group 2: 8-month median (OS)

ADT: androgen deprivation therapy; EBRT: external beam radiation therapy; LHRH: luteinizing hormone-releasing hormone; OS: overall survival; TUIP: transurethral incision of the prostate; TURP: transurethral resection of the prostate. Cytoreductive therapy includes open prostatectomy or TURP.
